# Methodology for the Regulation of Boom Sprayers Operating in Circular Trajectories

**DOI:** 10.3390/s110404295

**Published:** 2011-04-13

**Authors:** Francisco Javier Garcia-Ramos, Mariano Vidal, Antonio Boné, Alfredo Serreta

**Affiliations:** Superior Polytechnic School, University of Zaragoza, Spain; E-Mails: vidalcor@unizar.es (M.V.); anbone@unizar.es (A.B.); serreta@unizar.es (A.S.)

**Keywords:** boom, sprayer, GPS, circular trajectory, nozzle, flow control, surface dose, treatment uniformity

## Abstract

A methodology for the regulation of boom sprayers working in circular trajectories has been developed. In this type of trajectory, the areas of the plots of land treated by the outer nozzles of the boom are treated at reduced rates, and those treated by the inner nozzles are treated in excess. The goal of this study was to establish the methodology to determine the flow of the individual nozzles on the boom to guarantee that the dose of the product applied per surface unit is similar across the plot. This flow is a function of the position of the equipment (circular trajectory radius) and of the displacement velocity such that the treatment applied per surface unit is uniform. GPS technology was proposed as a basis to establish the position and displacement velocity of the tractor. The viability of this methodology was simulated considering two circular plots with radii of 160 m and 310 m, using three sets of equipment with boom widths of 14.5, 24.5 and 29.5 m. Data showed as increasing boom widths produce bigger errors in the surface dose applied (L/m^2^). Error also increases with decreasing plot surface. As an example, considering the three boom widths of 14.5, 24.5 and 29.5 m working on a circular plot with a radius of 160 m, the percentage of surface with errors in the applied surface dose greater than 5% was 30%, 58% and 65% respectively. Considering a circular plot with radius of 310 m the same errors were 8%, 22% and 31%. To obtain a uniform superficial dose two sprayer regulation alternatives have been simulated considering a 14.5 m boom: the regulation of the pressure of each nozzle and the regulation of the pressure of each boom section. The viability of implementing the proposed methodology on commercial boom sprayers using GPS antennas to establish the position and displacement velocity of the tractor was justified with a field trial in which a self-guiding commercial GPS system was used along with three precision GPS systems located in the sprayer boom. The use of an unique central GPS unit should allow the estimation of the work parameters of the boom nozzles (including those located at the boom ends) with great accuracy.

## 1. Introduction

The application of pesticides on agricultural crops requires that the dose of product applied per surface unit be adequate. Commercial sprayers are used for the application of phytosanitary products to achieve this goal. Sprayers are classified based on the crop used and treatment being implemented. Boom hydraulic sprayers, known as low crop sprayers, are mostly used in the specific case of extensive crops, such as corn, wheat, barley and tomato. Sprayers for low crops are comprised of one boom that has separate nozzles, each of which is set a fixed distance from the other. The height of the nozzles with respect to the crop must be adequate to guarantee a uniform product distribution.

When the trajectory followed by the sprayer during treatment follows a straight line, the surface area of the ground upon which each nozzle distributes the product is similar. However, this does not happen when the boom suffers horizontal speed variations [1] or when the sprayer trajectory is not straight. The edges of the plot are an example of not straight trajectories which can lead to overtreated or untreated areas, especially when equipment with a large boom width is used. Areas where spray overlap occurs could be reduced with map-based automatic boom section control [2].

When the equipment follows circular trajectories, such as those found in plots with center pivot irrigation systems, the lack of uniformity in chemical treatments can become unacceptable when the flow of each of the nozzles is the same. This is due to the nozzles on the outer part of the boom distributing the product over a greater area than those on the inner part. In this case, the areas affected by the outer nozzles receive a smaller amount of the product per surface area, leaving only part of the plot treated by default. In contrast, the areas treated by the inner nozzles have a greater amount of the product applied per surface unit, resulting in excess treatment. Therefore, it would be interesting, for circular trajectories, to have equipment with an adjustable flow for each of the nozzles in the boom. This would cause the inner nozzles to apply the product with a lower flow than those on the outer part. This could be achieved by controlling the flow from each nozzle and modifying it as a function of the circular trajectory radius and displacement velocity of the equipment so that the surface dose was uniformly applied. The use of this technology would avoid the presence of overtreated or untreated crop areas, ensuring that the crop receives the required dose (L/m^2^) to ensure the successful treatment of the plague.

Regarding this proposed methodology, equipment sets currently exist that have the technology to guarantee that the flow of each of the nozzles is proportional to the displacement velocity. This is achieved by varying the work pressure of the hydraulic circuit with the displacement velocity. The drawback to these systems is that all the nozzles have the same flow rate.

There is also technology to specifically control each nozzle by opening and closing systems [3–5], allowing the application of different doses to the surface. Another viable option involves the use of valves capable of individually regulating the flow of a single nozzle or a row of nozzles through the control of their work pressures [1,6,7]. In this respect, some manufacturers of boom sprayers have developed product flow control systems based on the utilization of pulse width modulation valves. Researchers [8] evaluated the effect of using commercially available controller systems (with automatic section control) in the nozzles pressure and uniformity. There were differences between auto-boom and auto-nozzle level control with each generating application errors varying in extent and magnitude.

On the other hand, the combination of GPS technology with variable product dosage application systems allows the optimization of the application of phytosanitary products. Researchers [9] designed and implemented a system for the differentiated application of herbicides using a boom sprayer. This system combined GIS information with the positioning of the application equipment using GPS in real time.

Agricultural tasks conducted using circular trajectories usually employ GPS technology to guarantee concentric patterns. This technology, applied to farm tractors, allows the instantaneous displacement velocity and the position of the equipment to be known [10,11]. Its application to plot courses with circular trajectories is a commercial reality and has been analyzed in different research studies for determining field efficiencies [12,13].

By combining the information provided by GPS technology (velocity and position of the machine) with the flow control systems of the nozzles on the boom, the application of uniform surface doses of pesticides can be guaranteed.

## 2. Experimental Section

The objectives of this work were to:

- Develop a calculation methodology that allows the estimation of, in the case of circular trajectories with boom sprayers, the flow of the individual nozzles on the boom to guarantee that the dose of the product applied per surface unit is similar across the plot.
- Analyze the uniformity of the surface dose considering two regulation systems: regulation of the pressure of nozzles and regulation of the pressure of boom sections.
- Verify the nozzle coverage areas calculated from a central GPS antenna by comparing these values to estimates from a GPS antenna located at a particular nozzle.

### 2.1. Calculation of the Uniformity of the Surface Dose Applied in Circular Trajectories

#### 2.1.1. Error Calculation of the Applied Surface Dose

The parameters that determine the dose applied by the hydraulic boom sprayers in straight-line trajectories are related according to Equation (1) [14]:

(1) $$\text{AV}=\frac{n\times \text{nfr}\times 60}{0.1\times n\times n_{s}\times \text{fs}}$$

where:

AV = application volume (L/ha)
n = nozzle number
nfr = nozzle flow rate (L/min)
n_s_ = nozzle separation (m)
fs = forward speed (km/h)

Based on Equation (1), a numerical calculation methodology was developed to calculate the lack of uniformity generated when boom sprayers are used in circular trajectories. The methodology developed to calculate the dose applied by a boom sprayer operating in circular trajectories is described as a function of the sprayer’s work radius and the selected nozzle. This methodology was developed assuming a boom equipped with nozzles of the same kind so that the flow contributed by each nozzle on the boom is the same.

The boom width (Bw) of the sprayer is defined as the distance (in meters) measured between the first and the last nozzle. Knowing the dose of product to be applied (L/ha) and considering a particular forward speed, the nominal pressure (p, measured in bars) must be selected according to the type of nozzle to apply the suitable nozzle flow rate (L/min) using Equation (1).

**Figure 1.**
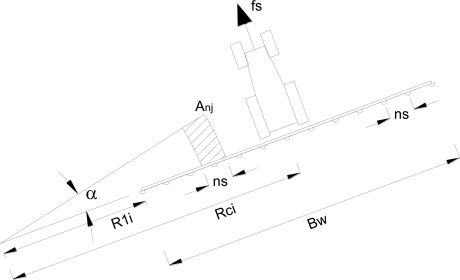
Work parameters of a boom sprayer following a circular trajectory. Rci = radius of the tractor axle to the center of the circumference i of the circular trajectory being considered; R1i = distance of the first nozzle on the boom to the center of the circumference plotted by the tractor on the i-th pass; Bw = width of the boom; ns = nozzle separation; α = angle traveled by the tractor per unit time; fs = displacement velocity of the tractor; and Anj = area treated by nozzle j per unit time assuming a work width of each nozzle with a value of ns and a linear displacement velocity of the tractor of s.

The height of the boom is that which, depending on the nozzle type, allows the necessary covering to obtain a uniform treatment of the work surface. Assuming that the equipment follows a circular trajectory with a work radius R_c_ (m) and considering any nozzle on the boom, the dose per surface unit for the area treated by each nozzle is obtained using the following parameters and equations (Figure 1), considering R_ci_ (m) as the radius of the tractor to the center of the circumference of the considered circular trajectory (calculated by the GPS system).

Angle traveled by the tractor per unit time (Equation (2)):

(2) $$\text{α}\;\left(\text{rad}/s\right)=\text{fs}\left(\text{km}/h\right)/\left(3.6\times R_{\text{ci}}\right)$$

Distance of the first nozzle on the boom (nozzle nearest to the center of the circular trajectory) to the center of the circumference plotted by the tractor on the i-th pass (Equation (3)):

(3) $$R_{\text{li}}\left(m\right)=R_{\text{ci}}-\left(\text{Bw}/2\right)$$

Position of each nozzle (j = 1, 2, ….n) as measured from the center of the circumference (Equation (4)):

(4) $$R_{j}\left(m\right)=R_{\text{li}}+[\left(j-1\right)\times n_{s}]$$

Area treated by each nozzle per unit time assuming a work width of each nozzle of n_s_ (Equation (5)):

(5) Anj(m2/s)=α/2×[(Rj+ns/2)^2−(Rj−ns/2)^2]

Surface dose (L/m^2^) applied by each nozzle (Equation (6)):

(6) $${\text{AV}}_{\text{nj}}\left(1/m^{2}\right)=\text{nfr}/\left(60\times A_{\text{nj}}\right)$$

Assuming that a treatment has to be administered using concentric circular trajectories, the applied surface dose will depend on the width of the work equipment for a treated surface. Knowing the work width of the equipment employed and fixing an application dose as an objective, the surface variation of the applied dose can be calculated for the different areas of the circular plot to be treated. Thus, based on the data from Equation (6), Equation (7) is developed to allow the determination of the percentage variation of the surface dose applied by each nozzle (AV_nc_ is the surface dose applied by the nozzle in the center of the boom):

(7) $$\Delta V\left(\%\right)=\left(\left({\text{AV}}_{\text{nj}}-{\text{AV}}_{\text{nc}}\right)/{\text{AV}}_{\text{nc}}\right)\times 100$$

#### 2.1.2. Simulation of the Work Realized with a Sprayer with a 14.5 m Wide Boom

As a practical example, the percentage variation of the surface dose applied by the nozzles of a boom sprayer with a 14.5-m-wide boom equipped with 30 nozzles each separated by 50 cm was considered.

Assuming that 240 L/ha must be applied, the nozzles used will have a pressure of 2 bar with a flow of 1.4 L/min, and the plot will be treated at a displacement speed of 7 km/h (according to Equation (1)).

The calculation methodology was developed using these conditions and assuming a circular plot of radius 160 m (neglecting the initial circular area corresponding to a radius of 10 m).

#### 2.1.3. Simulation of the Influence of the Work Width on the Variation of the Applied Surface Dose

A simulation analysis was performed on the variation of the surface dose applied by three sprayers, with boom widths of 14.5, 24.5 and 29.5 m (work widths of 15, 25 and 30 m) and nozzles separated by 50 cm, moving in circular trajectories in two plots with maximum radii of 160 and 310 m, respectively. The initial circular area corresponding to a radius of 10 m was neglected when carrying out the treatment. The radius of the treated surface and the work widths were chosen so that there was a whole number of circular trajectories traversed by the equipment to apply the product to both plots. A dose of 240 L/ha was fixed so that the nozzles had a nominal flow of 1.41 L/min and the equipment had a displacement velocity of 7 km/h.

### 2.2. Proposal for Sprayer Regulations to Obtain Uniform Surface Doses

#### 2.2.1. Methodology to Regulate the Nozzle Flow

Knowing the instantaneous position of the equipment along the circular trajectory (radius) and the instantaneous displacement speed (which requires the use of GPS technology), the area covered by each nozzle per unit time (A_nj,_ Equation (5)) and the dose to be applied, equivalent to that of the nozzle placed on the central position of the boom (AV_nc_, Equation (6)), can both be calculated. With these values, the necessary flow in each nozzle required to apply the same surface dose on the plot can be calculated (q_2i_, Equation (8)):

(8) $$q_{2j}\left(L/s\right)=A_{\text{nj}}\times {\text{AV}}_{\text{nc}}$$

Knowing the flow that each nozzle must contribute as a function of the position of the machine and its displacement velocity, the pressure at which each nozzle must operate to sustain such flow can be obtained using Equation (9):

(9) p2j(bar)=p×(60×q2j/nfr)^2

The regulation of the nozzle flow would translate into improvements in crop yield and would curb, in the case of over-application, waste in the application of chemical products, which would in turn yield a better economic bottom line [15], Considering under-application, it could produce reduced weed elimination which could result in crop yield loss due to weed competition.

Based on this proposal, individual pressure regulation systems could be established for each of the equipment nozzles to eliminate the lack of homogeneity in treatments that use circular trajectories with boom sprayers. Logically, this solution would require the individual control of each nozzle with the implementation of an individual pressure regulation system per nozzle, which would drive up the cost of the equipment. A second alternative would be to individually control the pressure in each boom section. With this case the necessary number of pressure regulation systems would be minor than in the individual nozzle control and would coincide with the number of sections of the sprayer boom. It must be considered that a boom section consists of several nozzles (4–8 commonly).

#### 2.2.2. Simulation of the Flow Regulation of a Boom Sprayer with 14.5 m Wide Boom

As a practical example, a boom sprayer with a 14.5-m-wide boom equipped with 30 nozzles each separated by 50 cm has been considered. The boom sprayer configuration considered is: 240 L/ha are to be applied at a pressure of 2 bar with a flow of 1.4 L/min, and a forward speed of 7 km/h.

Considering the sprayer configuration previously indicated, two regulation systems to obtain uniform surface doses have been analyzed: (a) the control of flow/pressure of boom sections of 5 nozzles (2.5 m width) or (b) the individual control of flow/pressure of each nozzle.

The calculation methodology was developed considering a circular plot of 160 m radius (neglecting the initial circular area corresponding to a radius of 10 m).

### 2.3. Comparison of the Surface Area Treated per Unit Time Using GPS Systems

**Figure 2.**
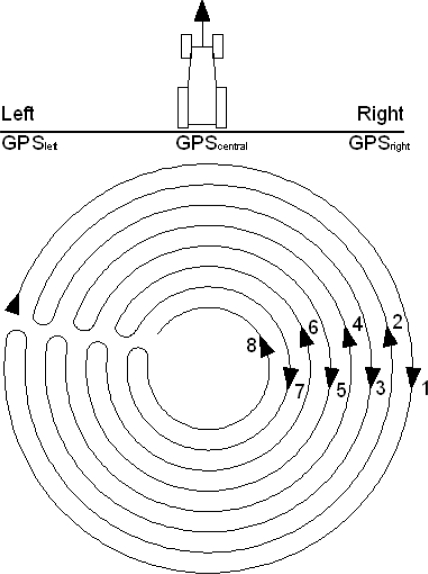
Field test performed in a plot with a boom sprayer that followed eight circular trajectories. The boom was equipped with three GPS systems with RTK correction and with a tractor with the commercial self guidance system John Deere Autotrac Universal, which was configured to make concentric pathways each separated by 16.75 m.

A field test was performed using a sprayer measuring 16.5 m, with 34 nozzles each separated by 50 cm (work width 17 m), that was connected to a tractor equipped with GPS John Deere Autotrac Universal with a SF1 correction (±33 cm precision). The tractor with an Autotrac Universal self guiding system was configured in the field to make concentric trajectories each separated by 16.75 m. A total of eight concentric passes were completed at a farm equipped with a center pivot irrigation system (Figure 2). The direction of the traversed surface was from the outer part of the plot to the inner part, such that trajectory 1 had the largest work radius, and trajectory 8 had the smallest work radius.

In addition, three Leica 1200 GPS systems with RTK correction (±2 cm precision; http://www.leica-geosystems.com/en/Leica-GPS1200_4521.htm) were installed on the boom. One of the systems was located on the center of the boom, and the other two were positioned on the left and right ends of the boom (Figure 1). The GPS antennas were fixed rigidly to the boom by means of metallic platens and screws. The interface of the GPS was introduced in a rucksack that was fixed to the boom, close to the antenna, by means of adhesive tape. The three GPS systems were configured to record data, with a sampling frequency of 1 s.

The concordance between the measurements made by the three GPS systems of the boom and by the tractor’s self-guidance GPS system was analyzed. On one hand, the error of the self-guiding system of the tractor when making concentric passes was analyzed. This was assessed by measuring the distance between the tractor’s axle during different concentric passes using data from the central GPS RTK. This distance was compared with the theoretical distance with which the GPS for the self guiding system of the tractor was configured (set as 16.75 m).

On the other hand, the viability of using an unique central GPS was analyzed comparing the estimation of the surface area treated per unit time by the nozzles located at the left and the right GPS RTK position. These values were obtained considering the data of the central GPS RTK and were compared to those obtained considering the GPS RTK located at each specific nozzle (left and right of the boom). For this goal, the radius of the tractor axle to the center of the circumference i (Rci) was calculated based on information provided by the GPS RTK located at the central position of the boom. With these data and knowing the width of the sprayer boom (Bw), the surface area treated by the two nozzles located in the same position as the left and the right GPS RTK on the boom were calculated using Equation (5). For those same nozzles, the surface area treated per unit time was calculated using the GPS located on the left and right positions of the boom.

## 3. Results and Discussion

### 3.1. Calculation of the Uniformity of the Applied Surface Dose Applied in Circular Trajectories

#### 3.1.1. Simulation of the Work Realized with a Sprayer with a 14.5 m Wide Boom

**Figure 3.**
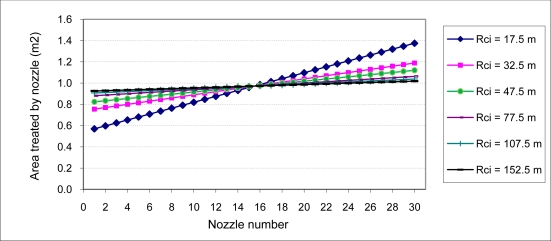
Area (m^2^) treated by each nozzle of a 14.5-m-wide boom with 30 nozzles each separated by 50 cm as a function of the circular trajectory radius (Rci). Nozzle 1 = inner nozzle; Nozzle 30 = outer nozzle.

**Figure 4.**
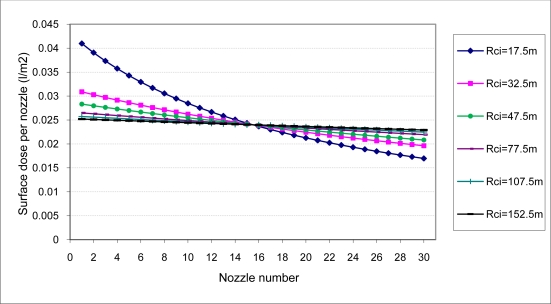
Surface dose (L/m^2^) applied by each nozzle on a 14.5-m-wide boom with 30 nozzles each separated by 50 cm as a function of the radius of the circular trajectory (Rci). Nozzle 1 = inner nozzle; Nozzle 30 = outer nozzle.

To develop the calculation methodology presented previously for each of the radii R_ci_ of the different circular trajectories needed to treat the entire plot, one would obtain (using Equation (5)) both the surface area treated by each nozzle on the boom (Figure 3) and (using Equation (6)) the dose of the product applied to each of those surfaces (Figure 4).

The theoretical surface upon which the flow of each nozzle must be distributed corresponds to that of a nozzle located at the center of the boom. As seen in Figure 3, the central point of the boom is the common intersection point of all the circular trajectories being considered.

In the same way, the theoretical surface dose that each of the nozzles should apply per surface unit corresponds to that of the nozzle located on the center of the boom that would be the same as the rest of the nozzles if the equipment was traveling in a straight line. Figure 4 shows how such doses are reduced in the outer nozzles and increased in the inner nozzles. This phenomenon becomes increasingly important in circular trajectories with a smaller work radius.

#### 3.1.2. Simulation of the Influence of the Work Width on the Variation of the Applied Surface Dose

**Figure 5.**
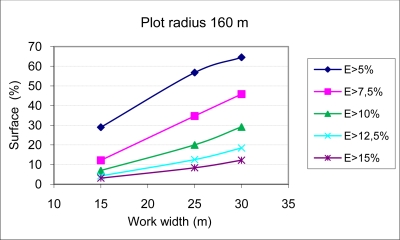
Percentage of surface area with errors (E) in the applied surface dose greater than 5%, 7.5%, 10%, 12.5% and 15% for the case of three sets of equipment with work widths of 15, 25 and 30 m carrying out treatment in circular trajectories in a circular plot with a 160 m-in radius at a displacement velocity of 7 km/h and a nominal nozzle flow of 1.4 L/min.

**Figure 6.**
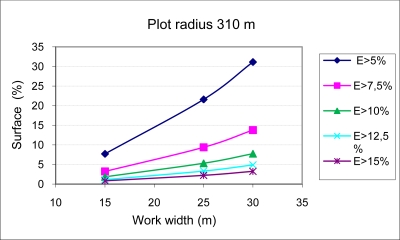
Percentage of surface area with errors (E) in the applied surface dose greater than 5%, 7.5%, 10%, 12.5% and 15% for the case of three sets of equipment with work widths of 15, 25 and 30 m carrying out treatment in circular trajectories in a circular plot with a 310 m-in radius at a displacement velocity of 7 km/h and a nominal nozzle flow of 1.4 L/min.

The results of applying equation 6 to two circular plots with radii of 160 m and 310 m, using three sets of equipment with boom widths of 14.5, 24.5 and 29.5 m (work widths of 15, 25 and 30 m), are shown in Figures 5 and 6. Figures 5 and 6 show for every boom width and plot size the percentage of the surface in which the surface dose has exceeded different values of percent variation of the fixed nominal surface dose to be applied as treatment (L/m^2^). With decreasing boom widths, there is an increasing homogeneity of treatment, while the applied dose per surface unit has a greater variability in equipment with large work widths. This phenomenon is of greater importance in plots with less surface area.

It is important to remember that even though the technical inspection of the application equipment is regulated by law in the majority of developed countries [16,17], in many cases, such legislation allows variation in the values of pressure and nozzle flow of up to 10%. This error would be compounded by that associated with circular trajectories, which can be very significant, as shown in Figures 5 and 6. With the methodology developed and knowing the plot to be treated and the equipment available, a precise determination can be made of the dose variation with respect to the fixed value through the calculation of the dose applied by each nozzle on the treated surface along its different concentric circular trajectories.

### 3.2. Proposal for Sprayer Regulations to Obtain Uniform Surface Doses

#### 3.2.1. Simulation of the Flow Regulation of a Boom Sprayer with 14.5 m Wide Boom

**Figure 7.**
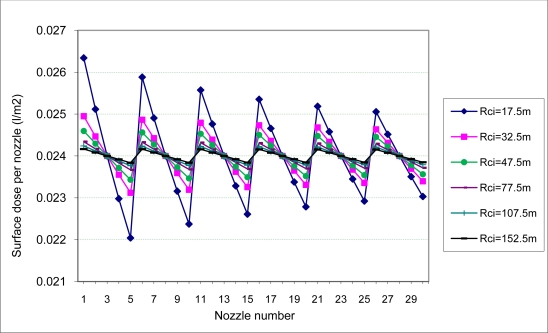
Surface dose (L/m^2^) applied by each nozzle on a 14.5-m-wide boom with 30 nozzles each separated by 50 cm as a function of the radius of the circular trajectory (Rci). Nozzle 1 = inner nozzle; Nozzle 30 = outer nozzle. The boom is divided in 6 sections of 5 nozzles. The pressure of each section is regulated in such way that the central nozzle of the section applies the required surface dose (0.024 L/m^2^).

**Control of Boom Sectors**. If the boom of 14.5 m width is sectioned in 2.5-m sections (five nozzles) to individually regulate the pressure of each section, the result would be equivalent to using aligned booms with a 2.5-m work width. The uniformity of the treatment increases with the decrease of the number of nozzles of the boom sections. The pressure of each boom section would be regulated considering Equations (8) and (9) so that the central nozzle of each boom section would apply the required surface dose. In this new situation, the variability in the dose applied per surface unit would drastically improve, as detailed in Figure 7 where the required surface dose per nozzle (0.024 L/m^2^) varies between 0.026 L/m^2^ (+9.75%) and 0.022 L/m^2^ (−8.16%) considering the boom section nearest to the center of the circular trajectory (nozzles 1 and 5) for the lowest tractor radius of 17.5 m.

If Figure 4 is analyzed under the same conditions, considering a boom without regulation, the required surface dose per nozzle (0.024 L/m^2^) varies between 0.042 L/m^2^ (+75.0%) for the nozzle 1 and 0.035 L/m^2^ (+45.8%) for the nozzle 5. As it is shown, the application uniformity improves for every trajectory if Figures 4 and 7 are compared.

Flow regulation considering boom sections allows an important improvement in the uniformity of the treatment. Considering a 14.5 m boom sprayer this improvement can be quantified by the comparison of Figures 4 and 7.

**Figure 8.**
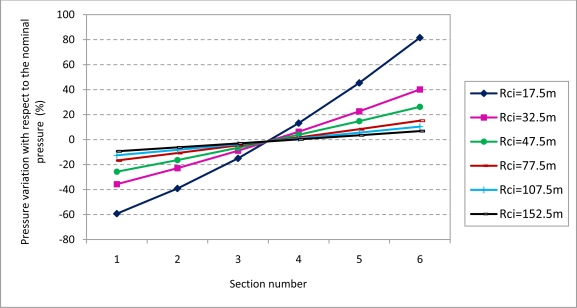
Percentage of pressure variation with respect to the nominal pressure considering the pressure regulation of each one of the 6 sections (5 nozzles per section) of a 14.5 m boom to obtain an uniform surface dose. Data have been obtained considering a 14.5 m boom sprayer carrying out treatment in circular trajectories in a circular plot with a 160 m—in radius at a displacement velocity of 7 km/h, and a nominal pressure of 2 bar (nozzle flow of 1.4 L/min).

The nozzle regulation pressure depends on the radius of the circular trajectory and on the position of the boom section. For instance, the maximum pressure variation (considering the nominal pressure of 2 bar) varies between 0.815 bar (−59.26%) and 3.632 bar (+81.58%) when the trajectory radius is 17.5 m (Figure 8).

**Figure 9.**
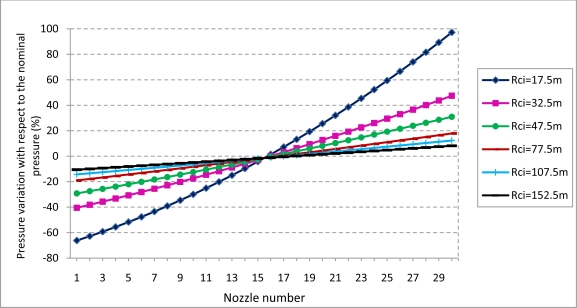
Percentage of pressure variation with respect to the nominal pressure considering the individual regulation of the nozzle pressure of a 14.5 m boom to obtain an uniform surface dose. Data have been obtained considering a 14.5 m boom sprayer carrying out treatment in circular trajectories in a circular plot with a 160 m-in radius at a displacement velocity of 7 km/h, and a nominal pressure of 2 bar (nozzle flow of 1.4 L/min).

**Control of Nozzles**. The individual control of the flow of each nozzle would guarantee an uniform superficial dose. To accomplish this, the work pressures of the nozzles must be modified individually on the basis of Equation (9). The nozzle pressure (considering the nominal pressure of 2 bar) varies between 0.676 bar (−66.18%) and 3.944 bar (+97.19%) when the trajectory radius is 17.5 m (Figure 9). The pressure variation decreases drastically with the increase of the trajectory radius. For instance, considering a trajectory radius of 32.5 m the nozzle pressure varies between 1.19 bar (−40.49%) and 2.95 bar (+43.79%). These pressure values are according to the pressure ranges admitted by the majority of the commercial nozzles used in boom sprayers.

**Control of Boom Sectors *versus* Control of Nozzles**. The individual control of nozzles, compared to boom sectors control, implies the absence of errors in the treatment uniformity because the pressure of each nozzle would be regulated to achieve the required surface dose. Application would be equivalent to a horizontal line of 0.024 L/m^2^ in Figure 7. However this solution produces a higher variation in the range of working pressures as it is shown comparing Figures 8 and 9, and consequently a bigger variation in the range of drop sizes. As the working pressure is reduced the drop volume median diameter increases [18]. Technically, the individual control of nozzles would require a specific valve per nozzle increasing the cost of the implementation of the control system. For the case of control of boom sectors it would be required a valve per sector.

Considering a boom of 14.5 m working in the conditions of Section 2.1.2, to guarantee the required surface dose of 0.024 L/m^2^ in whatever area of the plot, the work pressure should be regulated in such way that the outer nozzle (nozzle number 30) dose was the required dose (0.024 L/m^2^). In these conditions, considering the total area of the plot, the volume of product required for the treatment would be 2,086 L. Considering a boom with control of boom sectors of five nozzles (Figure 7), regulated to apply 0.024 L/m^2^ in the outer nozzle of each section the required volume would be 1,945 L (−6.75%), and for the case of a boom with individual control of nozzles the required volume would be 1,922 L (−7.86%). The reduction in the chemical consumption would justify the implementation cost of the selected control system. Besides, environmental contamination and over-treated areas would be decreased.

### 3.3. Comparison of the Surface Area Treated per Unit Time Using GPS Systems

**Figure 10.**
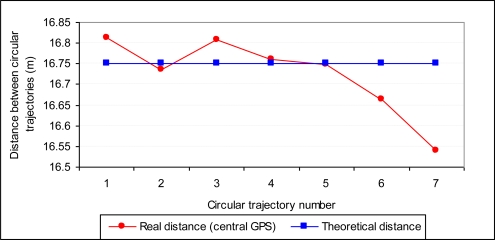
Real distance between concentric passes according to data from a GPS RTK located on the center of the boom in comparison to the theoretical distance programmed into the self-guidance system of the tractor equipped with a GPS John Deere Autotrac Universal.

The real median value of the distance between the circular trajectories obtained from the GPS RTK in the center of the boom was 16.7247 m, with a standard deviation of 0.095 m and a minimum-maximum range of variation of 16.54060 m–16.81220 m (Figure 10). If we compare these data with the programmed distance in the self guiding system of the tractor (16.75 m), the quality of the work performed can be concluded to be valid. In addition, the larger errors, as shown in Figure 10, were generated in passes six and seven, which were nearest to the center of the circular trajectories (smaller radius) and were, therefore, those in which the overlap between passes affected a smaller area of the plot. The sums of all the concentric distances were 117.25 m for the theoretical scenario and 117.07 m using the data provided by the GPS RTK located at the center of the boom.

For those same nozzles, the surface area treated per unit time was calculated using the GPS located on the left and right positions of the boom. The comparison data are shown in Table 1.

As shown, the data provided by the central GPS on the boom allow the estimation of the work parameters of the nozzles on the ends of the boom with great accuracy. The maximum percent error is −0.195% when comparing the real area measured with the GPS located over the nozzle itself with the area obtained from the data of the GPS located on the central part of the boom. Considering this information, it would be viable to establish a regulation system of the working parameters of the nozzles on the boom in real time based on the information supplied by the central GPS on the boom, with the error obtained as a function of the precision of the GPS equipment itself that, even in the case of a system without differential correction capabilities, would allow errors of around 6 cm, as shown in Figure 10.

It must be considered that the boom suffers horizontal and vertical movements during the field operations and consequently nozzle speed variations which produce irregularities in the spray distribution. A recent research [19] showed that, for a sprayer with a 22 m width boom working in field conditions at 9 km/h, the coefficient of variation of the boom speed considering all the nozzles was 4.5%. This error must be considered in the global analysis of the data because GPS data are affected by the boom movements. The lower variability of the data showed in Table 1 indicates a good compensation of the boom movements during the field test. However, further studies must be done using accelerometers located at the boom to estimate the influence of the boom movements on the GPS data.

The utilization of GPS technology would be profitable considering exclusively not overlapping between successive trajectories [17]. In addition, the sprayer regulation to apply uniform superficial doses will increase the profitability on having guaranteed the treatments success, increasing the crop production and quality and reducing the consumption of chemical products as it has been described at the end or Section 3.2.1.

## 4. Conclusions

Considering a boom sprayer working in circular trajectories regulated at a similar work pressure for each nozzle (similar flow rate), the superficial doses applied are reduced in the outer nozzles and increased in the inner nozzles. This phenomenon varies according to the boom width and the radius of the circular trajectory. The superficial dose has a greater variability in equipment with large work widths and becomes increasingly important in circular trajectories with a smaller radius.

A methodology has been developed to obtain the flow pressure required in every nozzle depending on the radius of the circular trajectory to apply uniform superficial doses. This methodology can be implemented in a commercial sprayer using GPS technology and pressure regulation systems.

A GPS located in the middle of the boom can be used to estimate the surface area treated by the different boom nozzles. The percentage of variation in the surface area treated by the outer and inner nozzles measured with GPS located at those nozzles varies a maximum of 0.092% compared to the surface area estimated for the same nozzles with a GPS located at the center of the boom.

To obtain a uniform superficial dose two sprayer regulation alternatives are proposed: the regulation of the pressure of each nozzle and the regulation of the pressure of each boom section. Considering individual nozzle regulation the treatment uniformity can be obtained by a major variability of flow pressure. Considering the boom section regulation the treatment uniformity increases with the decrease of the number of nozzles of the boom sections. In this case the pressure variability is lower than in the case of the individual regulation.

## Figures and Tables

**Table 1.** Surface area treated per unit time by the nozzles on either end of the boom calculated from the information provided by the GPS RTK located at the center of the boom and by the GPS RTK located on the right and left ends.

	**N° circular trajectory**	**m^2^/s estimated with central GPS RTK**	**m^2^/s measured with left GPS RTK**	**% variation**

Nozzle located on the left end of the boom	1	1.046	1.045	−0.092
	2	0.944	0.945	0.066
	3	1.068	1.067	−0.013
	4	0.932	0.933	0.091
	5	1.093	1.092	−0.07
	6	0.901	0.902	0.104
	7	1.130	1.131	0.035
	8	0.832	0.832	−0.005

	**N° circular trajectory**	**m^2^/s estimated with central GPS RTK**	**m^2^/s measured with right GPS RTK**	**% variation**

Nozzle located on the right end of the boom	1	0.945	0.945	0.005
	2	1.057	1.056	0.028
	3	0.941	0.942	−0.092
	4	1.077	1.077	0.029
	5	0.923	0.925	−0.195
	6	1.104	1.103	0.012
	7	0.876	0.877	−0.057
	8	1.168	1.169	−0.127

## References

[1] Lebeau F, El Bahir L, Destain MF, Kinnaert M, Hanus R (2004). Improvement of spray deposit homogeneity using a PVM spray controller to compensate horizontal boom speed variations. Comput. Electron. Agric.

[2] Luck JD, Pitla SK, Shearer SA, Mueller TG, Dillon CR, Fulton JP, Higgins SF (2010). Potential for pesticide and nutrient savings via map-based automatic boom section control of spray nozzles. Comput. Electron. Agric.

[3] Han S, Hendrickson LL, Ni B, Zhang Q (2001). Modification and testing of a commercial sprayer with PWM solenoids for precision spraying. Appl. Eng. Agric.

[4] Shahemabadi AR, Moayed MJ An algorithm for pulsed activation of solenoid valves for variable rate application of agricultural chemicals.

[5] Brown DL, Giles DK, Oliver MN, Klassen P (2008). Targeted spray technology to reduce pesticide in runoff from dormant orchards. Crop Protection.

[6] Kunavut J, Schueller JK, Mason PAC (2000). Continuous control of a sprayer pinch valve. Appl. Eng. Agric.

[7] Zhang X, Hu X, Mao W (2007). The Development of Intelligent Equipments about Precision Agriculture.

[8] Sharda A, Luck JD, Fulton JP, Shearer SA, McDonald TP Nozzle uniformity for agricultural sprayers operating under field operation when using automatic section technology.

[9] Al-Gaadi KA, Ayers PD (1999). Integrating GIS and GPS into a spatially variable rate herbicide application system. Appl. Eng. Agric.

[10] Schueller JK, Wanga M (1994). Spatially-variable fertilizer and pesticide application with GPS and DGPS. Comput. Electron. Agric.

[11] Gan-Mor S, Clark RL, Upchurch B (2007). Implement lateral position accuracy under RTK-GPS tractor guidance. Comput. Electron. Agric.

[12] Grisso RD, Jasa PJ, Rolofson DE (2002). Analysis of traffic patterns and yield monitor data for field efficiency determination. Appl. Eng. Agric.

[13] Grisso RD, Kocher MF, Adamchuk VI, Jasa PJ, Schroeder MA (2004). Field efficiency determination using traffic pattern indices. Appl. Eng. Agric.

[14] Weisser B, Koch H (2002). Expression of dose rate with respect to orchard sprayer function. Aspect Appl. Biol.

[15] Batte MT, Ehsani MR (2006). The economics of precision guidance with auto-boom control for farmer-owned agricultural sprayers. Comput. Electron. Agric.

[16] Ozkan E (1999). Recommendations for pesticide applicator training in USA based on licensing and training procedures in Western Europe. Appl. Eng. Agric.

[17] Gil E (2007). Inspection of sprayers in use: A European sustainable strategy to reduce pesticide use in fruit crops. Appl. Eng. Agric.

[18] Giles DK, Comino A (1990). Droplet size on spray pattern characteristics of an electronic flow controller for spray nozzle. J. Agric. Eng. Res.

[19] Ooms D, Lebeau R, Ruter R, Destain MF (2002). Measurements of the horizontal sprayer boom movements by sensor data fusion. Comput. Electron. Agric.

